# Mapping of Genetic Factors That Elicit Intermale Aggressive Behavior on Mouse Chromosome 15: Intruder Effects and the Complex Genetic Basis

**DOI:** 10.1371/journal.pone.0137764

**Published:** 2015-09-21

**Authors:** Aki Takahashi, Hiroki Sugimoto, Shogo Kato, Toshihiko Shiroishi, Tsuyoshi Koide

**Affiliations:** 1 Mouse Genomics Resource Laboratory, National Institute of Genetics (NIG), Mishima, Shizuoka, Japan; 2 Department of Genetics, SOKENDAI, Mishima, Shizuoka, Japan; 3 Laboratory of Behavioral Neuroendocrinology, University of Tsukuba, Tsukuba, Ibaraki, Japan; 4 Transdisciplinary Research Integration Center, Research Organization of Information and Systems, Minato-ku, Tokyo, Japan; 5 Division of Biology, Center for Molecular Medicine, Jichi Medical University, Shimotsuke, Tochigi, Japan; 6 The Institute of Statistical Mathematics, Tachikawa, Tokyo, Japan; 7 Mammalian Genetics Laboratory, NIG, Mishima, Shizuoka, Japan; Université de Bordeaux and Centre National de la Recherche Scientifique, FRANCE

## Abstract

Despite high estimates of the heritability of aggressiveness, the genetic basis for individual differences in aggression remains unclear. Previously, we showed that the wild-derived mouse strain MSM/Ms (MSM) exhibits highly aggressive behaviors, and identified chromosome 15 (Chr 15) as the location of one of the genetic factors behind this escalated aggression by using a panel of consomic strains of MSM in a C57BL/6J (B6) background. To understand the genetic effect of Chr 15 derived from MSM in detail, this study examined the aggressive behavior of a Chr 15 consomic strain towards different types of opponent. Our results showed that both resident and intruder animals had to have the same MSM Chr 15 genotype in order for attack bites to increase and attack latency to be reduced, whereas there was an intruder effect of MSM Chr 15 on tail rattle behavior. To narrow down the region that contains the genetic loci involved in the aggression-eliciting effects on Chr 15, we established a panel of subconsomic strains of MSM Chr 15. Analysis of these strains suggested the existence of multiple genes that enhance and suppress aggressive behavior on Chr 15, and these loci interact in a complex way. Regression analysis successfully identified four genetic loci on Chr 15 that influence attack latency, and one genetic locus that partially elicits aggressive behaviors was narrowed down to a 4.1-Mbp region (from 68.40 Mb to 72.50 Mb) on Chr 15.

## Introduction

There are large individual differences in aggression, a trait that is widely conserved within the animal kingdom. In humans, genetic factors have significant effects on individual differences in aggression, and estimates of the heritability of aggressiveness have ranged from 28% to 47% [1] and 56% for an antisocial personality [2]. Studies using gene-modification techniques have revealed more than 50 genes that are involved in aggressive behavior in mice (for review see [3–5]). However, in natural populations, the extent to which each gene/genetic locus can contribute to individual differences in aggression remains unknown. In addition, aggressive behavior itself has a complex nature as a social behavior in which the behavior of a test animal receives strong feedback from the behavior of other animals that it encounters [6,7]. Thus, when we study the genetic basis of aggressive behavior, it is always important to consider which aspect of such behavior is modulated by the genetic factor(s) in question.

Previously, we reported that the wild-derived mouse strain MSM/Ms (MSM) shows an escalated level of aggressive behavior, and MSM males not only showed a high level of territorial aggression in the resident-intruder test and the novel open-field test, but also killed their littermates and even a female pair-mate in their home cage [8,9]. By using a panel of consomic strains of mice established by crossing the highly aggressive MSM strain with a laboratory strain, C57BL/6J (B6), which shows low-to-moderate aggression, we attempted to identify chromosomes associated with the heightened aggression observed in MSM. A consomic panel is a useful tool to examine the complex genetic architecture of behavior [10], and we mapped two chromosomes, Chr 4 and Chr 15, involved in different aspects of aggression: MSM Chr 15 was shown to increase agitation and readily provoke aggressive behavior, whereas MSM Chr 4 was found to induce a maladaptive aspect of aggression because it was shown to be associated with injurious aggression towards a pair-mate female [9]. It is noteworthy that, in this previous study, all consomic strains showed a lower level of aggression than MSM. These results suggest that multiple genetic loci are involved in the heightened aggressiveness of MSM. However, only the B6 strain was used as an opponent to test the aggressive behavior of consomic strains, and it was possible that the B6 intruder triggered a low level of aggression from the resident male.

In this study, we focused on MSM Chr 15, which is associated with a reduction in the threshold at which aggressive behavior is initiated. We first examined the behavioral characteristics of aggressive behavior of Chr 15 consomic strains in detail by focusing on the effect of opponent type. We then attempted to narrow down the genetic locus/loci on Chr 15 that is/are involved in aggression by establishing a panel of subconsomic strains of Chr 15.

## Results

### Chr 15 derived from MSM increases inter-male aggression

**Fig 1:**
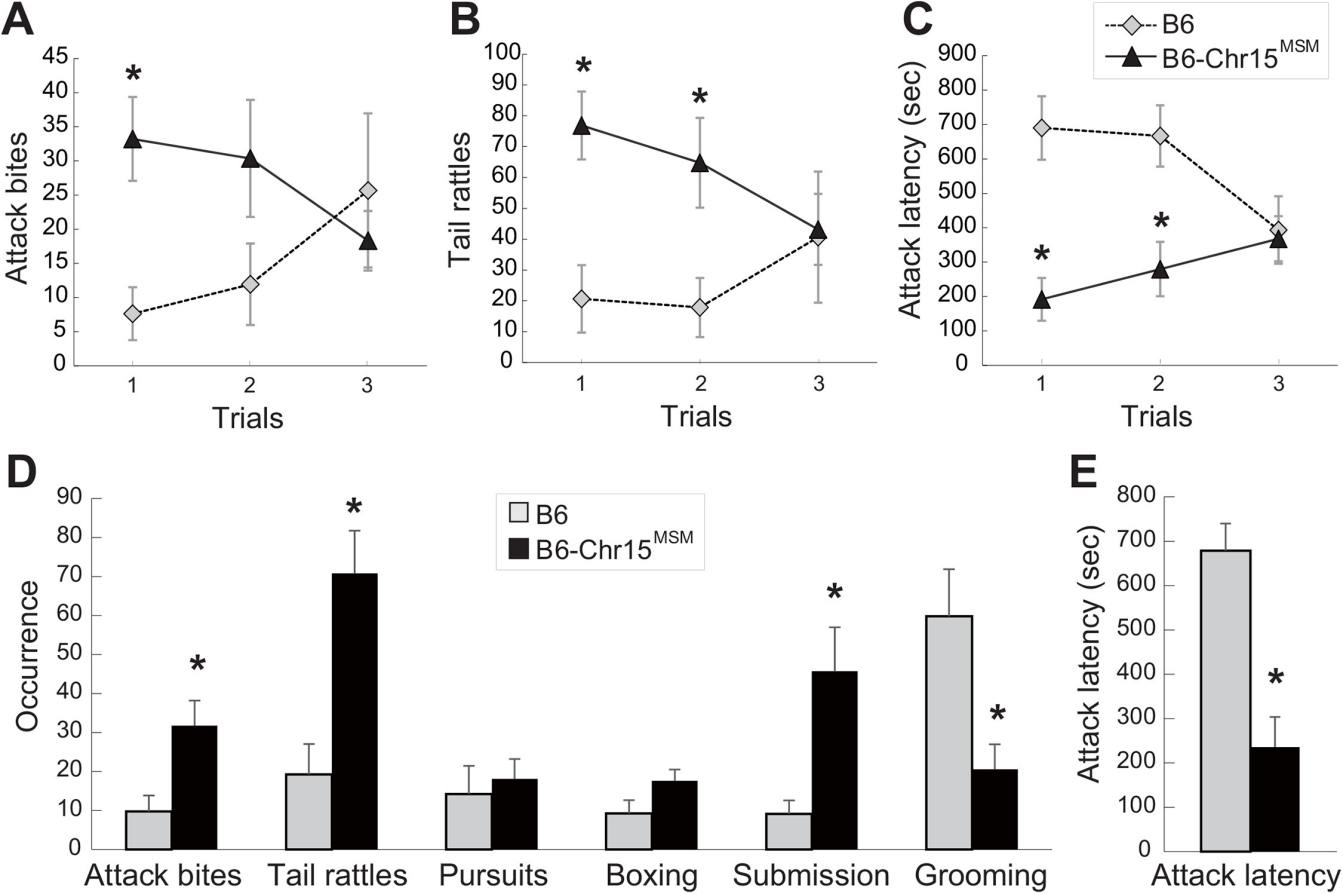
Aggressive behaviors in homogeneous set test. Aggressive behavior of B6 and B6-Chr15^MSM^ consomic strain in a homogeneous set test, in which males of the same strain were used as both residents and intruders. The trial-by-trial changes in attack bites [A], tail rattles [B], and attack latency [C] during three 15-min trials. B6 showed a gradual increase in aggressive behavior over the trials (dashed line), whereas B6-Chr15^MSM^ exhibited the opposite pattern (solid line). An asterisk indicates a significant strain difference when comparing corresponding trials (*p < 0.05). Average frequency of behavioral occurrences [D] and latency to first attack [E] in trials 1 and 2. An asterisk indicates a significant strain difference in each behavioral index (*p < 0.01). Error bars indicate standard error of the mean (SEM).

Previously, we reported that the B6-Chr15^MSM^ consomic strain showed a longer duration of tail rattles and a higher proportion of animals that initiated aggressive behavior, but the frequencies of attack bites and pursuits were similar to those in the B6 strain, when we used the B6 strain as an intruder [9]. In the present study, we used a modified version of the resident-intruder test (individuals with the same genotype as intruders, 10 days of isolation before the test) to obtain a clearer strain difference of aggressive behavior between B6 and B6-Chr15^MSM^. In a homogeneous set test (i.e., with individuals from the same strain), we found significant strain × trial interactions in attack bites (F(2,46) = 3.857, p = 0.028) and attack latency (F(2,46) = 7.007, p = 0.0022), and a trend in tail rattles (F(2,46) = 2.845, p = 0.068). The trial-by-trial pattern showed that B6 pairs exhibited low rates of attack bites and tail rattles, as well as long attack latency in their first trial, and the rates of these aggressive behaviors increased in successive trials (Fig 1A–1C). B6-Chr15^MSM^ pairs showed the opposite pattern: the most highly aggressive behaviors were found in trial 1 and became less intense thereafter. Thus, significant strain differences in aggressive behaviors were observed in the first two trials, but they had disappeared by trial 3. Therefore, we used the average data of trials 1 and 2 for further analyses. B6-Chr15^MSM^ pairs showed significantly higher occurrences of attack bites, tail rattles, and submissive posture (Fig 1D) and a shorter attack latency (Fig 1E) than B6. Furthermore, the number of animals that initiated aggressive behavior in trial 1 was higher in B6-Chr15^MSM^ (93%, 13 out of 14) than in B6 (31%, 4 out of 13). B6 showed a higher rate of grooming than B6-Chr15^MSM^ (Fig 1D).

### Intruder effects on aggression of Chr 15 consomic mice

**Fig 2:**
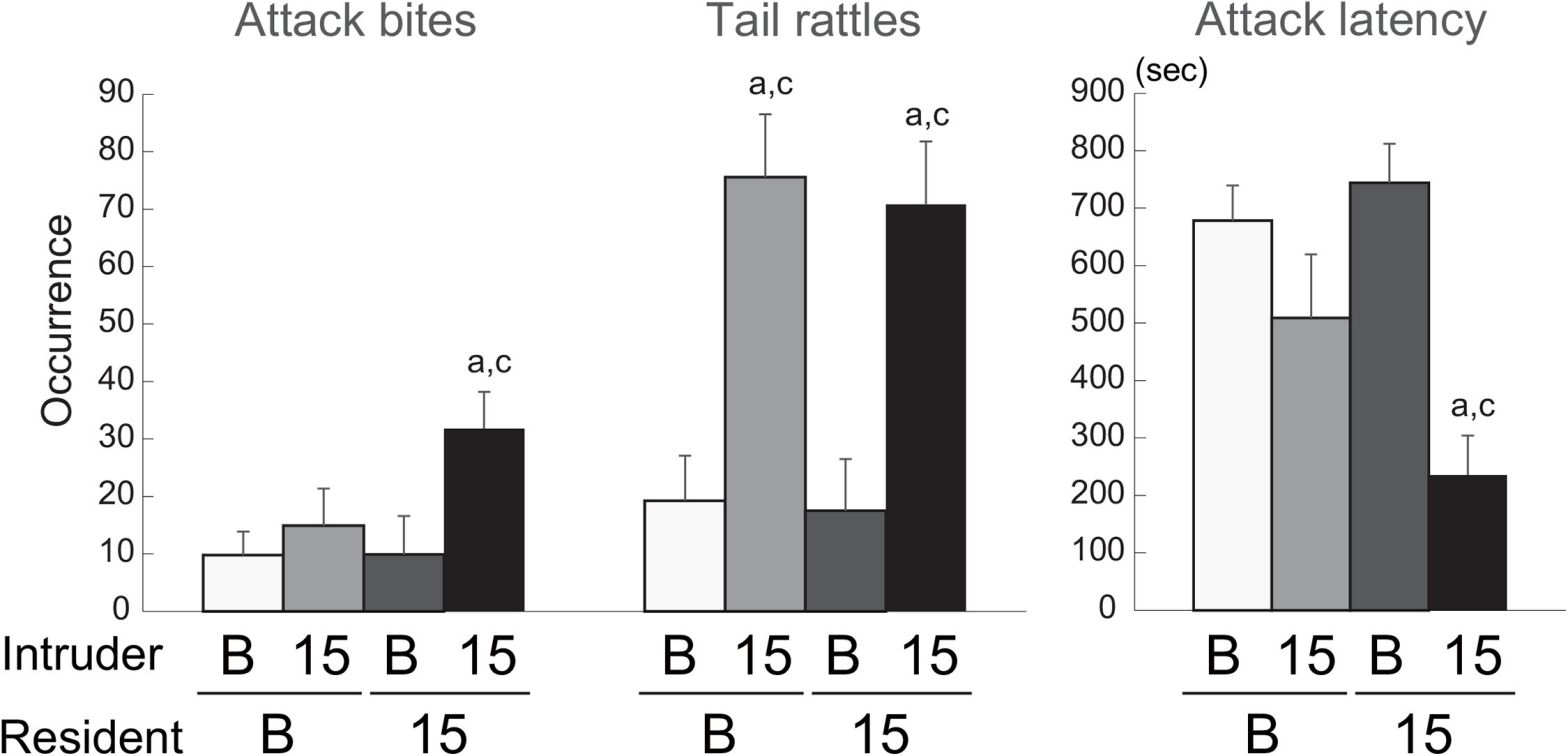
The effect of opponent’s genotype as observed in the heterogeneous set test in B6 and B6-Chr15^MSM^. The B6 (B) and B6-Chr15^MSM^ (15) residents encountered intruders of either genotype, so there were four types of encounter: B vs. B, B vs. 15, 15 vs. B, and 15 vs. 15 (resident vs. intruder, respectively). Strong effects of the opponent were observed in terms of attack bites, tail rattles, and attack latency. Data represent the average of trials 1 and 2, and error bars indicate SEM. a: p < 0.05 compared with B-B, b: p < 0.05 compared with B-15, and c: p < 0.05 compared with 15-B by Tukey-Kramer’s t-test. The data for the homogeneous pairs are from Fig 1.

We then examined the effect of the opponent’s genotype by a heterogeneous set test, in which B6 residents encountered B6-Chr15^MSM^ intruders (B-15) and B6-Chr15^MSM^ residents encountered B6 intruders (15-B). The results of the homogeneous set tests (B-B and 15–15) were combined with those of the heterogeneous set tests in order to conduct two-way ANOVA to examine the effect of the genotypes of both resident and intruder males. There was no effect of the resident’s genotype on any behaviors (Table 1). However, there were significant effects of the intruder’s genotype on attack bites (F(1,41) = 5.474, p = 0.024), tail rattles (F(1,41) = 16.215, p = 0.0002), and attack latency (F(1,41) = 19.222, p < 0.0001) (Fig 2). *Post hoc* test showed significant increases of tail rattles in B-15 pairs and 15–15 pairs, but not in 15-B pairs, compared with B-B pairs. A significant resident genotype × intruder genotype interaction was found only in attack latency (F(1,41) = 4.807, p = 0.034), and significantly shorter attack latency was observed in 15–15 pairs, but not in 15-B or B-15, compared with B-B. Although no significant interaction was detected, a similar pattern of strain differences was observed in attack bites and 15–15 pairs showed a significantly higher rate of attack bites than B-B, as determined by Tukey-Kramer’s t-test. These results suggest that the B6-Chr15^MSM^ males increased tail rattles when they were used as intruders regardless of the genotype of the resident, whereas attack bites were increased only when B6-Chr15^MSM^ residents encountered B6-Chr15^MSM^ intruders.

**Table 1:** Comparison of homogeneous and heterogeneous set tests.

Resident	B6	B6	15	15
Intruder	B6	15	B6	15
Attack bites	9.8±4.1	14.9±4.4	9.9±6.7	31.8±6.4 *
Tail rattles	19.2±7.8	75.6±24.9 *	17.5±9.0	70.8±11.0 *
Pursuits	14.2±7.2	8.6±3.8	9.4±7.2	18.1±5.1
Boxing	9.3±3.4	22.4±5.7	9.2±3.1	17.6±2.9
Submission	9.2±3.4	42.1±12.2	21.9±10.6	45.7±11.3
Grooming	59.8±15.0	37.1±8.9	51.0±18.1	20.6±7.2
Attack latency	678.3±61.2	508.8±110.7	744.3±67.9	235.7±68.3 *
% aggressive pairs	31%	55%	33%	93%
Total test pairs	13	10	10	14

Behavior of C57BL/6J (B6) and B6-Chr15^MSM^ consomic strain (15) in the homogeneous set test and the heterogeneous set test. Average frequencies of occurrence (by 1 sec time-sampling method) of each behavioral item in trials 1 and 2 are shown. For the percentage of aggressive pairs, that showed attack bites behavior, the result of trial 1 is indicated.

*p < 0.05 compared with B6 vs. B6 pair.

**Fig 3:**
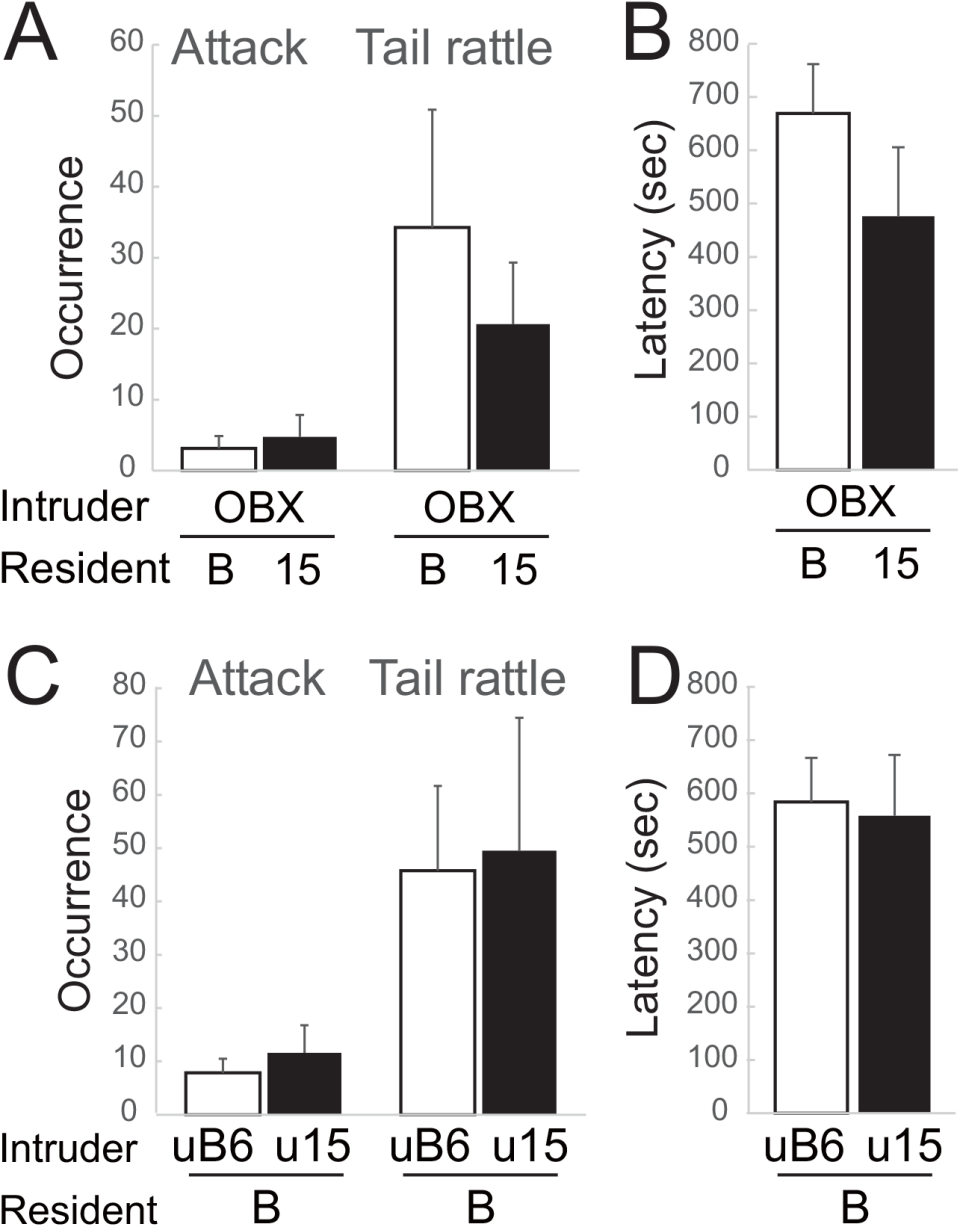
Aggressive behavior of B6 and B6-Chr15^MSM^ consomic strain towards OBX ICR intruders [A, B] or B6 intruders to which urine had been applied [C, D]. No strain differences were detected between B6 (B) and B6-Chr15^MSM^ (15) in terms of [A] the rates of attack bites and tail rattles, and [B] the latency to first attack towards OBX ICR intruders. In addition, no significant difference of urine pheromones between B6 and B6-Chr15^MSM^ males, which was examined by applying the urine of either group onto the backs of B6 intruders, was detected in terms of [C] the rates of attack bites and tail rattles, and [D] the latency to first attack. Error bars indicate SEM.

To determine the behavioral difference between B6 and B6-Chr15^MSM^ males as residents towards other types of common intruder, we performed the standard opponent test using olfactory bulbectomized (OBX) ICR/Jcl (ICR) males, which exhibit no aggressive behavior, as intruders. One-way ANOVA showed a significant strain difference only in grooming behavior (F(1,14) = 4.371, p = 0.047), and B6 residents showed significantly more grooming of OBX intruders than B6-Chr15^MSM^ residents (Table 2). We did not observe any significant strain difference between B6 and B6-Chr15^MSM^ males in any aggressive behaviors (Fig 3).

**Table 2:** Effects of OBX intruder mice on behaviors of the residents.

Resident	B6	15
Intruder	OBX ICR	OBX ICR
Attack bites	3.1±1.7	4.8±3.1
Tail rattles	34.25±16.6	20.6±8.7
Pursuits	0.5±0.5	0±0
Boxing	0.4±0.4	0±0
Submission	21.3±10.8	28.4±15.7
Grooming	73.0±19.9	26.3±8.2 *
Attack latency	669.4±92.3	476.1±129.7
% aggressive pairs	50%	63%
Total test pairs	8	8

Behaviors of the B6 and B6-Chr15^MSM^ (15) residents upon encountering an OBX ICR intruder. Average frequencies of occurrence (by 1 sec time-sampling method) for each behavioral item for trials 1 and 2 are shown. For the percentage of aggressive pairs, the result of trial 1 is indicated. An asterisk indicates a significant strain difference between B6 resident and 15 resident (p < 0.05).

Since we observed that B6-Chr15^MSM^ males caused an increase of tail rattles in the pair when they were used as intruders regardless of the genotype of the resident male, we next explored the cause of this “intruder effect” which elicits tail-rattle behaviors. We analyzed the behavior pattern of each resident and intruder male independently using the data presented in Fig 2 in order to identify whether B6-Chr15^MSM^ intruders show a high rate of tail rattles. However, there were no statistically significant differences in the frequencies of tail rattles, attacks, and grooming between residents and intruders in any pair-type (S1 Fig). We also examined which animal initiated tail rattles and again did not find any evidence that B6-Chr15^MSM^ intruders initiate tail rattles earlier than B6. Thus, behaviorally, we could not dissect the cause of this intruder effect observed in B6-Chr15^MSM^.

It has been reported that urinary pheromones are among the most effective factors to trigger aggressive behavior in mice [6,11,12,13]. Therefore, we next examined the role of urine pheromones in the intruder effect of B6-Chr15^MSM^. Urine was collected from B6 and B6-Chr15^MSM^ males, and B6 intruder males had the urine of either strain applied to their backs using a cotton swab before the resident-intruder test. There was no significant effect of urine type on any behavioral measurements (Fig 3 and Table 3). However, it should be noted that there were increases in tail rattles and the percentage of animals that initiated attacks when we used intruders to which urine had been applied (either B6 or B6-Chr15^MSM^ urine) compared with non-urine-treated B6 intruders (Table 1), suggesting that the smell from the intruder could affect the resident’s aggressive behavior.

**Table 3:** Effects of urine on behaviors of the residents.

Resident	B6	B6
Intruder (Urine)	B6 (B6)	B6 (15)
Attack bites	7.9±2.6	11.6±5.2
Tail rattles	45.8±15.9	49.5±24.9
Pursuits	1.9±1.1	2.6±1.1
Boxing	6.2±2.0	7.3±3.2
Submission	9.6±3.7	17.4±8.0
Grooming	41.6±9.1	37.8±11.9
Attack latency	558.0±82.7	584.3±114.4
% aggressive pairs	88%	63%
Total test pairs	8	8

Behaviors of the B6 residents upon encountering an intruder to which urine had been applied. Urine was collected from either B6 males or B6-Chr15^MSM^ (15) males.

### Genetic mapping of loci for aggressive behavior on Chr 15

**Fig 4:**
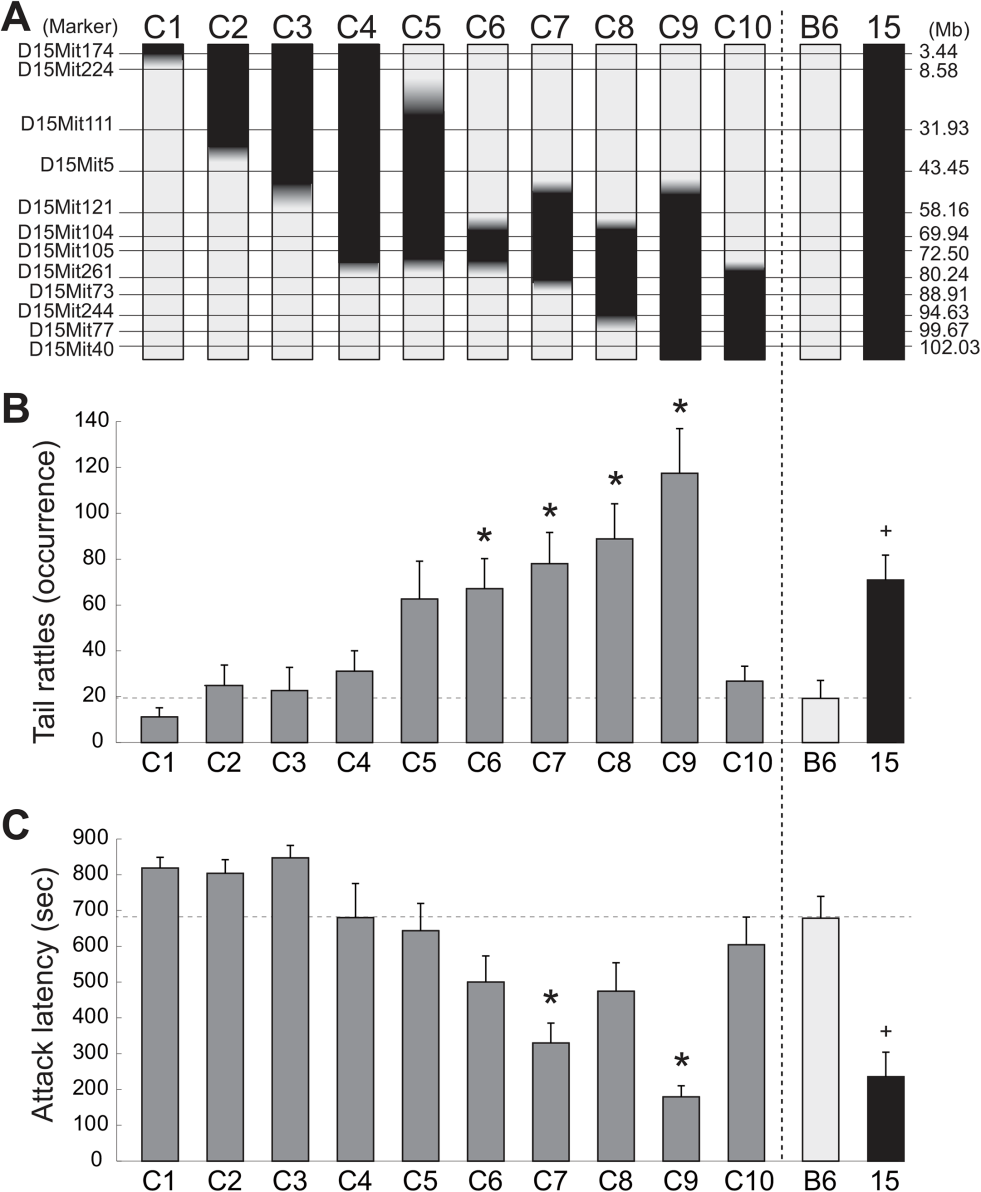
Establishment and analysis of a panel of subconsomic strains of MSM Chr 15. [A] Diagram of subconsomic strains used for the homogeneous set test. The horizontal lines indicate the approximate locations of genetic MIT markers, and their physical positions (Mb) are indicated. Chromosomal regions that were substituted with DNA from MSM are shown in black. The average frequency of occurrence of tail rattles [B] and the latency to the first attack [C] for trials 1 and 2 in 10 subconsomic lines during the resident-intruder test are shown. An asterisk indicates a significant difference when compared with B6 (*Dunnett’s t-test, p < 0.05). The result of B6-Chr15^MSM^ (15) is indicated as a reference (data from Fig 1, +p < 0.05). Error bars indicate SEM.

To identify genetic locus/loci on Chr 15 that evokes/evoke aggressive behavior in males, we established 10 lines of subconsomic strains to encompass the whole chromosome (Fig 4A). A homogeneous set test was used to examine their aggressive behavior because B6-Chr15^MSM^ increased attack bites only when they encountered intruders with the same genotype. There were significant effects of strain on attack bites (F(10,110) = 5.242, p < 0.0001), tail rattles (F(10,110) = 8.319, p < 0.0001), submissive posture (F(10,110) = 12.189, p < 0.0001), and attack latency (F(10,110) = 10.600, p < 0.0001) (Table 4). Dunnett’s t-test indicated that subconsomic strains C6, C7, C8, and C9 showed significantly higher rates of tail rattles than B6 (Fig 4B). Meanwhile, latency to the first attack was significantly decreased in C7 and C9 at the similar level as B6-Chr15^MSM^ compared with that in B6 (Fig 4C).

**Table 4:** Aggressive and non-aggressive behaviors of a panel of subconsomic strains of B6-Chr15^MSM^ in the homogeneous set test.

Resident	C1	C2	C3	C4	C5
Intruder	C1	C2	C3	C4	C5
Attack bites	0.6±0.2	2.3±1.1	2.3±1.4	6.0±3.0	3.9±1.1
Tail rattles	11.1±4.1	24.9±8.9	22.7±10.1	31.1±8.9	62.6±16.5
Pursuits	0.1±0.1 *	1.2±0.7 *	2.1±1.5	2.3±1.6	3.4±2.1
Boxing	1.0±0.4	1.5±0.8	6.9±3.7	5.3±2.2	2.6±1.0
Submission	1.0±0.6	8.3±5.2	5.7±4.4	3.0±1.3	21.4±7.6
Grooming	68.0±15.5	49.9±10.8	50.5±9.8	59.7±15.6	75.8±20.7
Attack latency	818.8±30.1	804.0±38.2	847.2±34.8	680.0±95.3	643.6±76.5
% aggressive pairs	50%	50%	27%	36%	90%
Total test pairs	12	10	11	11	10
Resident	C6	C7	C8	C9	C10
Intruder	C6	C7	C8	C9	C10
Attack bites	9.3±2.9	16.3±4.4	16.0±4.1	22.4±3.6 *	4.5±1.5
Tail rattles	67.1±13.1 *	78.0±13.6 *	88.8±15.3 *	117.5±19.5*	26.8±6.6
Pursuits	4.2±1.8	6.9±2.7	9.5±3.7	8.5±2.0	2.5±1.4
Boxing	7.0±1.8	9.3±2.7	5.2±2.2	8.3±2.3	3.4±2.0
Submission	15.4±3.7	22.0±5.3	29.3±9.1	79.8±12.1 *	21.2±7.0
Grooming	84.4±7.0	60.1±14.3	87.5±12.8	79.1±14.1	80.5±19.0
Attack latency	500.2±73.1	329.8±55.8*	474.2±80.1	179.5±30.9*	604.3±77.4
% aggressive pairs	83%	100%	91%	100%	70%
Total test pairs	12	11	11	10	10

The average frequency of occurrence of each behavioral item in trials 1 and 2 is shown. For the percentage of aggressive animals, the result of trial 1 is indicated.

*p < 0.05 compared with B6 vs. B6 pair.

**Fig 5:**
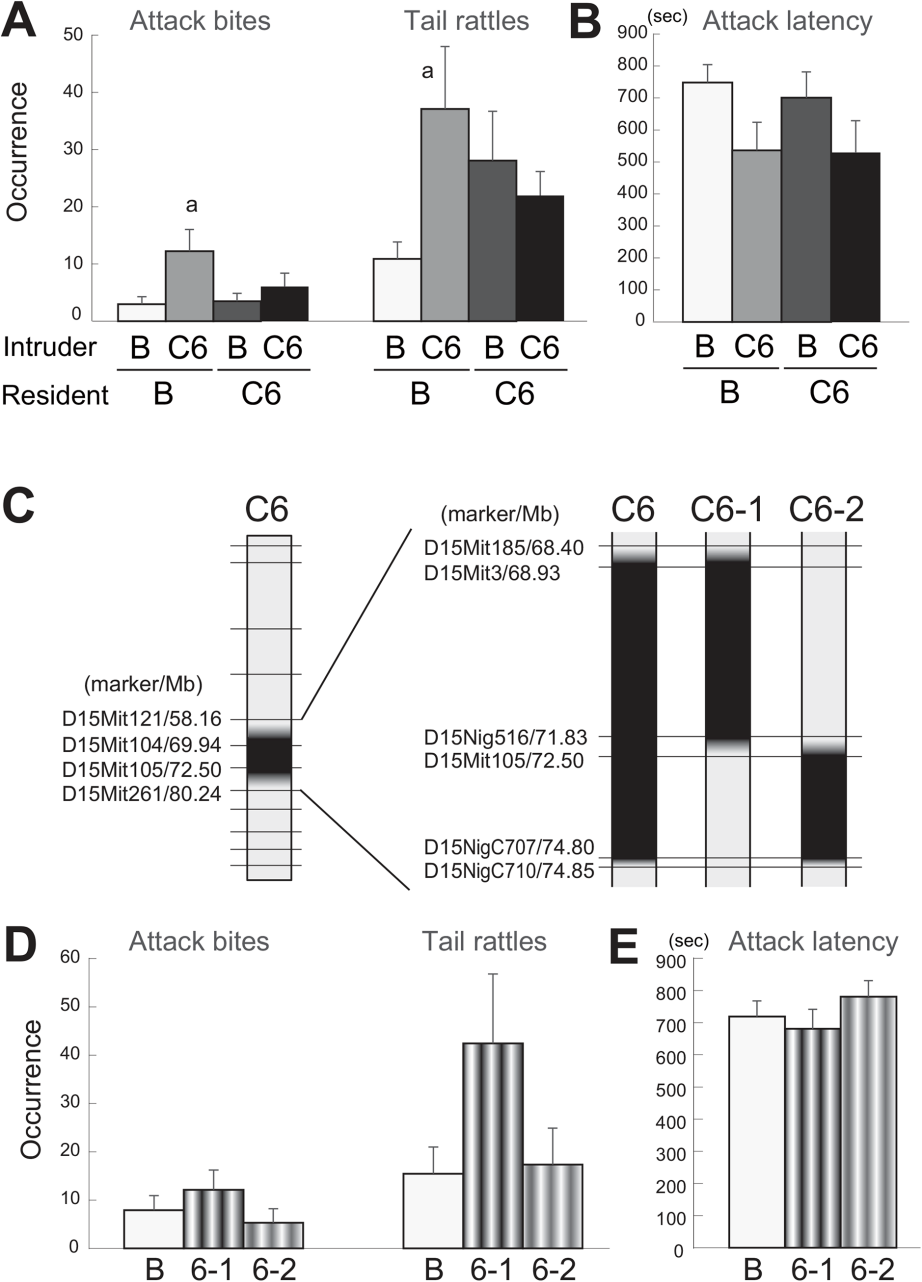
Analyses of intruder effects and genetic loci. Detailed analysis of the C6 subconsomic strain in terms of the intruder effect [A, B] and further mapping of the genetic locus [C, D, E]. The effect of the opponent’s genotype was also observed in the heterogeneous set test using the C6 subconsomic strain [A, B]. The B6 (B) and C6 subconsomic strain (C6) residents encountered intruders with either genotype, so there were four types of encounter: B vs. B, B vs. C6, C6 vs. B, and C6 vs. C6 (resident vs. intruder, respectively). The C6 intruder caused increases of attack bites and tail rattles [A], and a shortening of attack latency [B]. A diagram of two sub-subconsomic strains from the C6 subconsomic strain [C]. The genetic markers that were used for the establishment of these strains are indicated. Chromosomal regions that were substituted with DNA from MSM are shown in black. The frequencies of occurrence of attack bites and tail rattles [D], and the latency to the first attack [E] are indicated. An asterisk indicates a significant difference when compared with B6 (t-test with Bonferroni’s correction, p < 0.05). a: p < 0.05 compared with B-B, b: p < 0.05 compared with B-C6, and c: p < 0.05 compared with C6-B by Tukey-Kramer’s t-test.

Although there were decreases in aggressive behavior in strains with increasingly small sections of the C9 MSM region, the C6 subconsomic strain, which had the shortest MSM region, still showed a significantly high level of tail rattles in the homogeneous set test. Thus, we expect that one of the candidate genes which is involved in the aggression eliciting effect of B6-Chr15^MSM^ is located within the C6 region. To examine whether this locus also possesses the “intruder effect” observed in the parental B6-Chr15^MSM^ males, we tested C6 subconsomic strains in the heterogeneous set test (Fig 5A and 5B). New sets of animals were used for both homogeneous set groups (B-B and C6-C6) and heterogeneous set groups (B-C6 and C6-B), and two-way ANOVA was conducted to examine the effect of the genotypes of both resident and intruder males. There was no significant effect of the resident’s genotype on any behavior. However, there were significant effects of the intruder’s genotype on attack bites (F(1,46) = 7.659, p = 0.0081) and attack latency (F(1,46) = 5.416, p = 0.024), confirming an important role of this genetic locus on the intruder effect. For the tail rattles, there was a significant resident genotype × intruder genotype interaction (F(1,46) = 7.022, p = 0.011). *Post hoc* test showed significant increases of attack bites and tail rattles in B-C6 pairs compared with B-B pairs (Fig 5A). However, there were no significant differences in aggressive behavior between C6-B and C6-C6 pairs.

For fine mapping of the C6 region, we established two sub-subconsomic strains, C6-1 and C6-2, from the C6 subconsomic strain (Fig 5C). Further genotyping of Chr 15 gave an estimate of the physical distance of the C6 region, ranging from 68.23 Mb to 74.63 Mb, with C6-1 and C6-2 sub-subconsomic strains each possessing around half of this region. We conducted the homogeneous set test on C6-1 and C6-2 males, as well as B6 males (Fig 5D and 5E). One-way ANOVA showed a significant trend of genotype for tail rattles (F(2,68) = 2.425, p = 0.096), but not for attack bites or attack latency. There was a trend of an increase of tail rattles in the C6-1 sub-subconsomic strain, but this did not reach statistical significance due to large variance (t(48) = 1.859, p = 0.069; without any correction for multiple testing). The C6-1 strain also showed a higher occurrence of attack bites (Fig 5D) and slightly shorter attack latency compared with B6 (Fig 5E), but the differences were small and not statistically significant. By contrast, the C6-2 strain was similar in all behavioral indices to B6.

**Fig 6:**
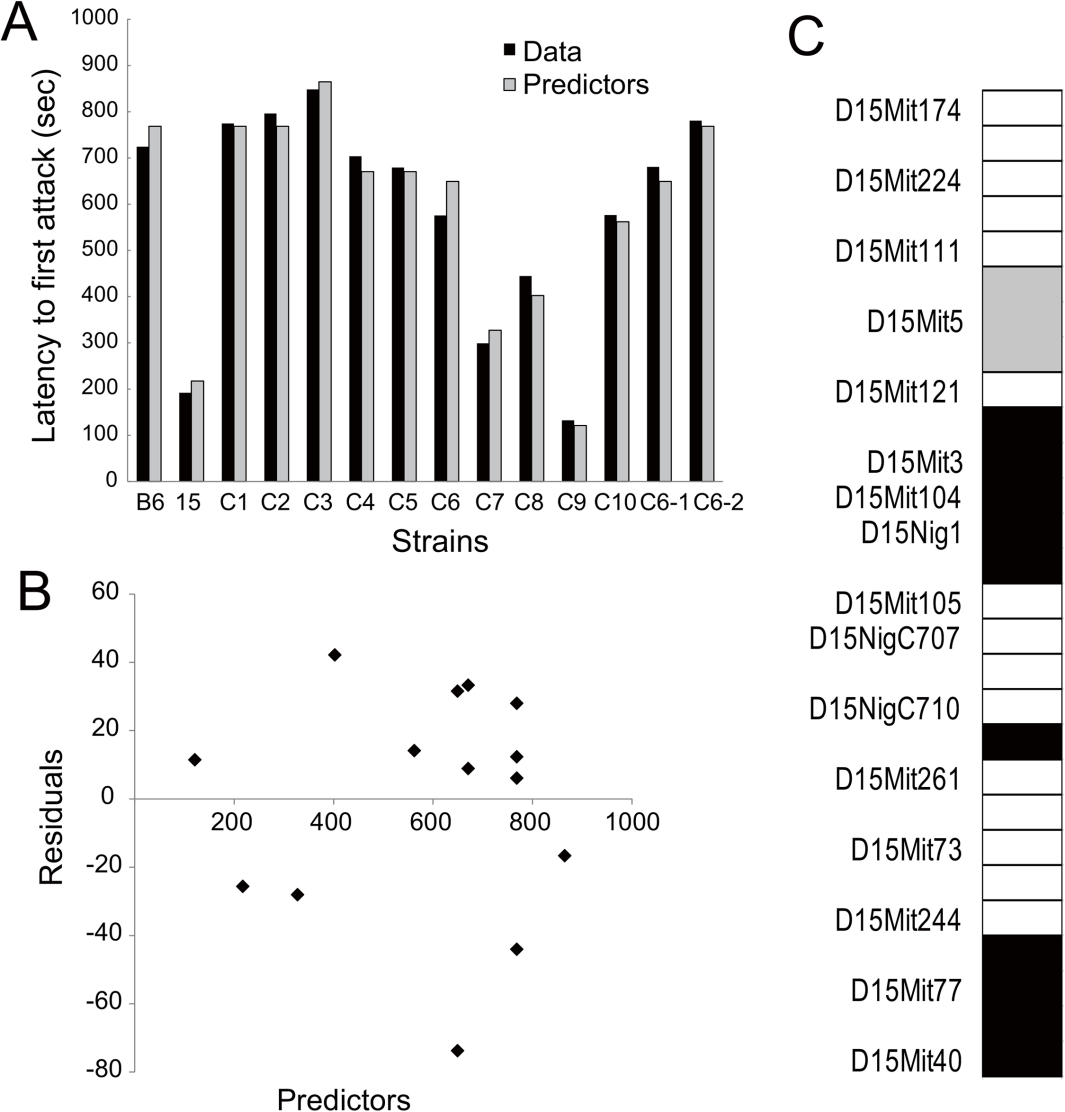
The least squares linear regression model identified four genetic loci on Chr 15 for the attack latency. [A] The predictors estimated by the regression model fitted to the actual data for each strain. [B] A plot of the residuals and predictors showed no inter-correlations, suggesting the independence of the model errors. [C] The mapped location of genetic loci by the regression model for attack latency on Chr 15. Black areas are the loci that decrease attack latency, and the gray area indicates the locus that increases attack latency.

To identify genetic loci that are involved in aggression-eliciting effects on Chr 15 statistically, we used a regression method that was previously adapted to map genetic loci for home-cage activity in subconsomic strains of Chr 6 [14]. This method considers an additive effect of each locus as well as an interactive effect between adjacent loci, but does not consider the interaction between loci located far apart. We used the behavioral data of 14 strains and the genotype data of 16 microsatellite markers as well as 15 interactions between the adjacent markers in this analysis. After pretreatment to reduce redundant variables, 14 out of 31 variables remained for the least absolute shrinkage and selection operator (lasso) (Table 5). Then, we conducted the lasso, followed by an analysis of all subsets to detect loci with a statistically significant effect on each behavior. Our analysis showed a reasonable fit of the model to attack latency, but not to tail rattles and attack bites. For the attack latency, the lasso (with estimated tuning parameter lambda = 2.22) reduced the number of variables from 14 to 8. The total amount of variance, which measures how far the residuals of the fitted model are spread out, was 945.68. Then, we fitted the least squares linear regression model to the data whose regressors were those selected by the lasso (Table 5). The number of selected variables now became four, and the maximum log-likelihood was -68.40 and the value of Akaike Information Criterion (AIC) was 150.80. The total amount of variance was 1025.785, which is 8% more than that estimated by the lasso. Residual analysis to compare the predicted values (predictors) of the model and the data showed a reasonable fit of this model (Fig 6A), and the largest value of residuals (-73.74) observed in the C6 strain was only 12.8% of the measured value (575.45) of attack latency in this strain (S1 Table). In addition, there was no clear relationship between predictor and residuals (Fig 6B), suggesting that the model provided a satisfactory fit to the data for the attack latency. From the results of our regression analysis, we successfully mapped four genetic loci for attack latency on Chr 15 (Fig 6C). Regression coefficients suggested that there are three genetic loci that decrease attack latency and also one genetic locus that increases it on MSM Chr 15. The three loci that shorten the attack latency were mapped between D15Mit3 and D15Nig1 (estimate = -119.145, p = 0.007), between D15NigC710 and D15Mit261 (estimate = -246.815, p < 0.001), and between D15Mit244 and the telomere (estimate = -206.268, p < 0.001). The genetic locus that prolongs the attack latency was mapped between D15Mit111 and D15Mit5 (estimate = 96.377, p = 0.018).

**Table 5:** Estimates of the regression coefficients for attack latency.

Regressors (markers)	Estimated coefficients		p-value
	Lasso	All subsets	
Intercept (β0)	757.1	768.3	1.51×10^−10^
D15Mit174	0.9	*	
D15Mit224 (:174,: 111)	0.0		
D15Mit111	38.6	*	
D15Mit5(:111)	42.1	96.4	**0.018222**
D15Mit5: D15Mit121	0.0		
D15Mit121(:3)	-49.0	-75.1	0.102486
D15Mit3—D15Nig1	-76.5	-119.1	**0.007178**
D15Nig1: D15Mit105	-49.2	*	
D15Mit105—D15NigC707	0.0		
D15NigC710(:C707)	0.0		
D15NigC710: D15Mit261	-242.4	-246.8	**0.000149**
D15Mit261	0.0		
D15Mit73(:261)—D15Mit244	0.0		
D15Mit77(:244)—D15Mit40	-195.7	-206.3	**0.000226**

The ‘intercept’ of the regressors denotes the regression coefficient β0 of the regression models. The notation ‘A: B’ refers to the interaction between markers A and B, while ‘C—D’ represents the chromosomal region ranging from C to D. For the lasso, all of the markers are listed. Estimates of the regression coefficients and the corresponding p-values of the linear model (all subsets) are also presented. The significant p-values are indicated in boldface (p < 0.05). Asterisks represent the variables eliminated by all subsets.

By contrast, the same analyses on tail rattles and attack bites revealed much greater variance of the residuals (S1 Table). Therefore, this model, which does not consider inter-loci interaction between loci that are far apart on the same chromosome, did not fit well to the data for these behavioral measurements.

## Discussion

In this study, we first confirmed the aggression-promoting effect of MSM Chr 15 using a modified version of the resident-intruder test that employed animals with the same genotype as the resident and the intruder after 10 days in isolation. In line with a previous report that laboratory mice infrequently demonstrate aggressive behavior at the first encounter [15,16], we observed a gradual increase in aggressive behavior over the course of the trials in the B6 strain. By contrast, B6-Chr15^MSM^ exhibited the highest level of aggression at the first encounter, after which its aggressive behavior gradually decreased. Thus, B6-Chr15^MSM^ and B6 showed no differences in their aggressive behavior in the third trial. A possible explanation for this temporal pattern observed in B6-Chr15^MSM^ is that this strain experiences strong excitement at the first encounter and this state of over-arousal may be connected with heightened aggression [7]. As the animals became habituated to the test situation in the successive trials, it is possible that their high level of excitement was reduced and their aggression decreased to the same level as that of B6.

The nature of the opponent is one of the most important factors in aggressive behavior [6,7,17,18]; accordingly, in this study, we observed a strong effect of the intruder’s genotype on aggressive behaviors. Aggression-promoting effects of MSM Chr 15 on the frequency of attack bites and the attack latency appeared upon encountering individuals with the same genotype (B6-Chr15^MSM^), but disappeared upon encountering a B6 intruder or an OBX ICR intruder. By contrast, we observed that the pairs in which B6-Chr15^MSM^ was the intruder exhibited an increase of tail rattles regardless of the genotype of the resident male. These findings suggest that the genetic loci for attack bites/attack latency and tail rattles are different even within Chr 15. It is also likely that the genetic loci on Chr 15 are involved in factors eliciting aggression from an opponent, rather than enhancing attack behavior of the resident himself. This “intruder effect” observed for tail rattles did not seem to be dependent on the strain difference of the urine pheromones between B6 and B6-Chr15^MSM^. However, we found that the occurrence of tail rattles towards B6 individuals applied with urine (from an individual of either genotype) was higher than that towards untreated B6, suggesting that the smell of urine may affect the tail rattle behavior. We did not examine and compare the intensity of the smell between B6 and B6-Chr15^MSM^ males in this study, or the animals’ ability to discriminate between the odor of urine from those strains, so the possible involvement of urine pheromones remains to be examined in the future. Tail rattles have been considered to be stress-related behavior [19], and the intruder effect observed in our study could be a reflection of high social anxiety or excitement towards the intruder in the novel encounter situation. We previously examined the emotionality of these consomic strains and found that B6-Chr15^MSM^ showed high autonomic reactivity in the novel open-field test [20]. However, we did not observe a high level of tail rattles or other behaviors in B6-Chr15^MSM^ intruders compared with those in resident males in the present work. There may be a very subtle behavioral difference in B6-Chr15^MSM^, such as in terms of ultrasonic vocalization or the respiration pattern [21,22], which could not be examined in this study. Although further analysis is required to identify the component that influences the intruder effect in B6-Chr15^MSM^, our results indicate that the analysis of social behavior between animals of the same genotype provides a more sensitive way to detect the effects of genetics on aggression.

In a few studies, attempts have been made to map quantitative trait loci (QTL) that affect aggressive behaviors [9,18,23–25]. In addition to our study, another study also mapped genetic loci for aggression on Chr 15, as well as Chr 5 and Chr 10, using F_2_ individuals by crossing BALB/cJ and A/J mouse strains, focusing on attack latency toward a dangled intruder male [24]. Their interval mapping showed that the confidence interval for the location of the QTL was between 41.1 Mb and 112.5 Mb of Chr 15. In order to identify the precise location of the genetic locus associated with the aggression-eliciting effects of Chr 15 in our study, we established a panel of subconsomic strains of Chr 15 and analyzed their aggressive behavior using a homogeneous set test. However, our results showed marked complexity of the genetics of the aggression-promoting effect, even within a single chromosome. There were gradual increases in the frequency of tail rattles and attack bites as well as decreases in the attack latency in association with expansion of the substituted MSM region on the telomeric half of the Chr 15 (the results for subconsomic strains C6, C7, C8, and C9). The C9 strain that has the substituted MSM region covering the entirety of the telomeric half of Chr 15 (58.17 Mb to the telomere) showed the highest level of aggressive behaviors, so it is likely that there are multiple genetic loci within the C9 region that are involved in elevating aggressive behavior. This area overlaps with a QTL for attack latency identified in an F_2_ cross of BALB/cJ and A/J [24], so a genetic factor in the C9 region has been shown to be involved in agitation and a readiness to initiate/elicit aggressive behavior in multiple studies. Since the C4 strain (centromere to 72.53 Mb) and the C10 strain (80.29 Mb to telomere) did not show any behavioral difference from B6, but these strains possess a large overlap of the MSM region with highly aggressive strains (C6 to C9), it is possible that there is/are genetic locus/loci in the vicinity of the proximal and distal ends of this region that cancel out the effect of aggression-evoking loci.

Although our behavioral analysis suggested distinct genetic factors that elicit tail rattles and that promote attack bites and reduce attack latency, analysis of our subconsomic strains showed that there are different genetic bases but also large overlaps between those factors. The subconsomic strains that changed tail rattles, with a few exceptions, also changed attack behaviors (latency and frequency). By using regression analysis with a model that did not take into consideration distally located inter-locus interaction, we successfully mapped the genetic loci for the factors that change attack latency but not for the factors that elicit tail rattles. This suggests that there are complex inter-locus interactions within Chr 15 in the genetic factors that elicit tail rattle behavior, and thus we could not map the loci using our current model. Previous QTL study showed different genetic bases for tail rattles and attack latency [18], suggesting that these behavioral phenotypes reflect different aspects of aggression/emotionality.

Given that the C6 strain has the smallest MSM region with a moderate but significant effect on tail rattles, one of the genetic loci that elicit tail rattles was mapped between 68.40 Mb and 74.85 Mb of Chr 15. We showed that the C6 strain also exhibited the intruder effect in the form of B6-Chr15^MSM^ in the heterogeneous set test. Thus, there must be a gene at this locus that is the cause of the aggression-promoting effect of an intruder. To narrow down the location of the locus further, we established two sub-subconsomic strains and examined their aggression. Our results showed a trend of an increase of tail rattles relative to B6 in the C6-1 sub-subconsomic strain, which has the proximal segment of the C6 region, whereas no behavioral change was observed in the C6-2 strain, which has the distal segment of the C6 region. Our regression analysis also indicated that a genetic locus that decreases attack latency is located in the C6-1 region. Therefore, one of the genetic loci that affects aggressive behavior of male mice was successfully narrowed down to between 68.402 Mb (D15Mit185) and 72.500 Mb (D15Mit105) of Chr 15. In this 4.1-Mb range, there are 20 predicted genes, three of which have been identified as protein-coding genes: khdrbs3 (KH domain containing RNA binding signal transduction associated 3), fam135b (family with sequence similarity 135 member B), and col22a1 (collagen type XXII alpha 1). There are also some non-coding RNAs, so further study will be required to identify the polymorphism and mechanism that cause the increase in aggressive behavior.

Meanwhile, we noted attenuation of the aggression phenotype in association with a reduction in the size of the replaced region containing the target loci. The C9 strain that has the bottom half of Chr 15 from MSM showed the strongest aggression-promoting effect, and the shortening of the MSM region caused step-by-step reductions of this effect, with the C6-1 strain with the smallest region of the MSM chromosome ending up being only slightly more aggressive than B6. This complex effect is not uncommon in this kind of study [14,26–29]. A recent meta-analysis study, which reviewed more than a hundred human genetic association studies, failed to find genetic polymorphisms that are associated with aggression outcome at the 5% level of significance [30]. This may have been due to the involvement of multiple small-effect genes on aggression with complex gene-environmental interaction [31] and gene-gene interaction [32], as has been reported as “missing heritability” [33]. Even in our mouse study with the same genetic background, our results showed a quite complicated genetic architecture on Chr 15 that is involved in the factors that elicit aggressive behaviors of male mice. Further analysis is required to understand the genetic regulation of the aggression-promoting effect of B6-Chr15^MSM^, and this phenomenon should provide insight into the “missing heritability” of aggression.

## Materials and Methods

### Animals

B6-Chr15^MSM^ consomic strains had been established at the National Institute of Genetics (NIG), Mishima, Japan, by back-crossing MSM/Ms (MSM) into C57BL/6JJcl (B6) over 10 generations and a final inter-crossing to obtain a pair of MSM Chr 15 on the genetic background of B6 [34]. In this study, a series of subconsomic strains of MSM Chr 15 were established from B6-Chr15^MSM^ by back-crossing B6-Chr15^MSM^ into B6 for two or more generations, and the offspring that carried a desirable recombination within Chr 15 were finally intercrossed to make homozygotes for the substituted segment (see the “Establishment and analysis of subconsomic strains” section). B6 and ICR/Jcl were purchased from CLEA Japan, Inc. (Tokyo, Japan). All animals were maintained at the NIG under a 12/12-hour light/dark cycle (lights on from 08:00 to 20:00) in a temperature-controlled room (23 ± 2°C). Test males were weaned from their parents at around 3 to 4 weeks of age and housed in same sex groups until preparation of the behavioral tests. All tests were carried out from 16:00 to 20:00. Food and water were available ad libitum. This study was carried out in strict accordance with the recommendations in the Guidelines for Proper Conduct of Animal Experiments of the Science Council of Japan. The protocol was approved by the Institutional Committee for Animal Care and Use of the NIG (Permit Numbers: 23–10, 24–10, and 25–10). All surgery was performed under sodium pentobarbital anesthesia, and all efforts were made to minimize suffering.

### Behavioral analysis

#### Resident-intruder test

Males that were naïve to any behavioral test were used at the age of 10 weeks. In this study, two types of encounter, homogeneous and heterogeneous, were examined. 1) In the homogeneous set test, individuals with the same genotype were designated as resident and intruder: B6-Chr15^MSM^ vs. B6-Chr15^MSM^ (number of residents, n = 14) and B6 vs. B6 (n = 13). 2) In the heterogeneous set test, the resident and intruder differed in terms of their genotype: B6-Chr15^MSM^ vs. B6 (n = 10) and B6 vs. B6-Chr15^MSM^ (n = 10).

Ten days before the resident-intruder test, each mouse was weighed and the heavier individual was assigned to be the resident male. Then, both resident and intruder animals were housed individually until the test; the resident animal was housed in a large home cage (22 × 32 × 13.5 cm) and the intruder animal was housed in a small cage (14 × 35 × 13.6 cm) with wood chips as bedding material. In the homogeneous set test, littermates were used for the same resident-intruder encounter. However, when the size of the litter was small, or for the heterogeneous set test, individuals from different litters of similar ages were used as the intruders. In the resident-intruder test, the experimenter introduced an intruder into the cage of the resident mouse, and their behaviors were videotaped for 15 min. After the test, the intruder male was removed from the resident’s cage and returned to his home cage. This aggressive encounter was repeated three times (one test per day on three consecutive days) using different intruder males.

#### Standard opponent test with OBX ICR intruder

In this test, we used OBX ICR males as intruders against both B6 and B6-Chr15^MSM^ resident males because it has been shown that OBX males rarely show aggressive behavior but have the ability to elicit aggressive behavior from an opponent because their gonads are intact [7]. Ten days before the test, ICR males were olfactory bulbectomized under anesthesia. After recovery from this surgery, OBX mice were group-housed six males per cage, and individuals who did not show any aggressive behavior in their home cage after the operation were used as intruders. ICR males of approximately 8-weeks-old were used as the intruders against 11- to 12-week-old resident males of either B6 (n = 8) or B6-Chr15^MSM^ consomic mice (n = 8). The test procedure of the standard opponent resident-intruder test was the same as in the heterogeneous set test, and aggressive behavior was observed for 15 min. In this experiment, only one aggressive encounter was conducted.

#### Urine swab test

To examine the role of pheromones contained in urine on aggressive behavior in B6-Chr15^MSM^, we examined the effect of B6-Chr15^MSM^-derived urine on aggressive behavior. Urine was collected from 10 animals per strain during the early phase of the light cycle. Since animals tended to urinate when held in the hand, we held animals above a glass plate to collect their urine. Urine mixtures of either B6-Chr15^MSM^ or B6 were stored at 4°C for up to one month. In the behavior test, a new set of B6 males were used as both residents and intruders, and eight resident males were examined for their aggression against B6 intruders to which urine from B6-Chr15^MSM^ had been applied, along with eight resident males for B6 intruders to which urine from B6 had been applied. Immediately before the resident-intruder test, 60 μl of the urine mixture was absorbed by cotton wool, which was applied around the neck and the upper base of the tail of the intruder mouse. Each resident male was tested twice with the same intruders to which urine had been applied.

#### Quantification in resident-intruder test

In this study, the behaviors of both resident and intruder during an aggressive encounter lasting 15 min were observed from video recordings by human observers. The behavioral items included the following six behaviors as described by Abeelen (1964) with a 1-sec time-sampling method [35]: attack bites: biting the opponent; tail rattles: flicking the tail often with a pounding sound; pursuit: one mouse chases after the other; boxing: offensive upright posture displayed in both mice concurrently; submission: rearing up on the hind legs, drawing the forelegs close to the body or extending them stiffly and remaining motionless; and grooming: grooming the fur of the other mouse or mounting it. We also analyzed the attack latency, defined as the time until the first attack bite. For behavioral analysis of the C6 subconsomic strain and C6 sub-subconsomic strains, we analyzed only three indices: the occurrences of attack bites and tail rattles, and the attack latency.

### Establishment and analysis of subconsomic strains

B6-Chr15^MSM^ mice were crossed with B6 to produce the F_1_ generation, and backcrossed with B6 for one to two generations to obtain meiotic recombinants. Several recombinants that carry different parts of MSM-derived Chr 15 were bred to B6 in order to obtain pairs that have the same recombination segment of MSM. By intercrossing those mice, we obtained homozygotes of the recombinants and established a panel of subconsomic strains of Chr 15. The 10 subconsomic lines were established to encompass the whole chromosome. The following genetic MIT microsatellite markers were used for genotyping during the establishment of the subconsomic strains (Mouse Microsatellite DataBase of Japan, MMDBJ, NIG): D15Mit174 (3.44 Mb), D15Mit224 (8.58 Mb), D15Mit111 (31.93 Mb), D15Mit5 (43.45 Mb), D15Mit121 (58.16 Mb), D15Mit104 (69.94 Mb), D15Mit105 (72.50 Mb), D15Mit261 (80.24 Mb), D15Mit73 (88.91 Mb), D15Mit244 (94.63 Mb), D15Mit77 (99.67 Mb), and D15Mit40 (102.03 Mb) (position information obtained from the Mouse Genome Informatics database (MGI)). To obtain sub-subconsomic strains, we backcrossed the C6 strain into B6 to obtain a recombinant within the C6 region. For the genotyping to establish sub-subconsomic strains of C6, we added two MIT markers, D15Mit185 (68.40 Mb) and D15Mit3 (68.93 Mb), as well as three additional markers, D15Nig516 (71.83 Mb, F: AAAAAGGATGGGCTTTTCTT, R: GGAGAATGGAAAGGAAGAGG), D15NigC707 (74.80 Mb, F: GGTGACCTCCAAGAAATTGGAATG, R: CATGTAGAAGCCAGAGTTATGC), and D15NigC710 (74.85 Mb, F: GTCCTGGCCACTGTCTCAGCA, R CAGGCCTGGGAAGTGAGGT), which we custom-made in order to detect microsatellite polymorphisms between B6 and MSM by referring to the NIG Mouse Genome Database (http://molossinus.lab.nig.ac.jp/msmdb/index.jsp). Genomic DNA of each animal was prepared from the tail or ear and polymerase chain reaction (PCR) was used to detect sequence length polymorphisms between MSM and B6. Such polymorphisms were detected by agarose gel electrophoresis with 3% agarose in 1 × TAE buffer, visualized by ethidium bromide staining. For the behavioral analysis of the panel of subconsomic strains and sub-subconsomic strains, we conducted the homogeneous set test, as described above.

### Statistical analysis

Data analysis was performed using StatView version 5 (SAS Institute Inc.). Two-way ANOVA was conducted to examine the trial-by-trial change of aggressive behaviors in B6 and B6-Chr15 MSM, and to examine the effect of the genotypes of the resident and the intruder in the homogeneous and heterogeneous set tests. One-way ANOVA was conducted to examine the effect of the genotype of residents paired with OBX ICR male intruders, the effect of the genotype of individuals who were the source of urine applied to B6 intruders, and the effect of genotype in the panel of subconsomic strains or sub-subconsomic strains. In the case of a significant F value, Tukey-Kramer’s t-test was conducted in the homogeneous and heterogeneous set tests in order to compare each pair-type. For the analysis of subconsomic strains and sub-subconsomic strains, we conducted Dunnett’s t-test to detect significant strain differences in comparison with B6 in subconsomic strains and sub-subconsomic strains.

### Mapping of genetic loci with regression models

In order to map multiple loci that influence aggressive behaviors, we applied a method of genetic mapping using regression models, which we reported previously [14]. For the analysis using regression models, the behavioral data and the genotype data for 14 strains including subconsomic strains (C1-C10), sub-subconsomic strains (C6-1 and C6-2), and parental strains (B6 and B6-Chr15^MSM^) were used. Three regression models were established in which the responses are the behavioral data on tail rattles, attack bites, and attack latency. The regressors of all of the models were commonly assumed to be the genotype data of 16 microsatellite markers as well as 15 interactions between the adjacent markers of each strain. In order to eliminate redundant regressors used for regression analysis, the markers that showed the same genotype as the adjacent markers for all of the strains were recognized as one marker (variable). First, we performed model selection via the least absolute shrinkage and selection operator (lasso) [36]. As a next step, the least squares linear regression model was applied to the data in which the regressors are those selected via lasso. In order to select variables further, we adopted all subsets based on the AIC. The subset that minimized the AIC value was ultimately used for fitting [37]. Estimates of the regression coefficients and the corresponding p-values were obtained. Residual analysis was conducted to assess the goodness-of-fit of the model. The regression analysis was performed by using the statistical software R. To estimate the regression coefficients and tuning parameter of the lasso, the R package ‘glmnet’ was used. The generic function ‘lm’ was used for estimation of the least squares linear regression model.

## Supporting Information

S1 Fig Aggressive behavior of either resident or intruder animal in homogeneous and heterogeneous set tests in B6 and B6-Chr15^MSM^. The B6 (B) and B6-Chr15^MSM^ (15) residents encountered intruders with either genotype, and thus there were four types of encounter: B vs. B, B vs. 15, 15 vs. B, and 15 vs. 15 (resident vs. intruder, respectively). The data were re-analyzed from Fig 2. Error bars indicate SEM.
(EPS)

S1 Table The predictors and residuals estimated using all subsets for each strain. (DOCX)
