# Supplementary material for: Mapping of Genetic Factors That Elicit Intermale Aggressive Behavior on Mouse Chromosome 15: Intruder Effects and the Complex Genetic Basis
Source: PLoS One. 2015 Sep 21;10(9):e0137764. doi: 10.1371/journal.pone.0137764 (PMC4577130; doi:10.1371/journal.pone.0137764)
Supplement: S1 Table — (DOCX) [file pone.0137764.s002.docx]

**Supporting Information**

**S1 Table.** The predictors and residuals estimated using all subsets for each strain.

|  | Attack latency | | | Tail rattles | | | Attack bites | | |
| --- | --- | --- | --- | --- | --- | --- | --- | --- | --- |
| Strain | Predictors | Residuals |  | Predictors | Residuals |  | Predictors | Residuals |  |
| B6 | 768.3 | -44.0 |  | 15.8 | 0.3 |  | 2.1 | 4.8 |  |
| B6-15MSM | 217.3 | -25.6 |  | 82.5 | -5.6 |  | 28.6 | 4.6 |  |
| C1 | 768.3 | 6.1 |  | 15.8 | -1.6 |  | 2.1 | -1.3 |  |
| C2 | 768.3 | 28.0 |  | 15.8 | 5.8 |  | 2.1 | 0.0 |  |
| C3 | 864.7 | -16.6 |  | 15.8 | 11.3 |  | 2.1 | -0.1 |  |
| C4 | 670.4 | 33.3 |  | 50.4 | -14.2 |  | 8.1 | -2.6 |  |
| C5 | 670.4 | 8.9 |  | 50.4 | 19.8 |  | 8.1 | -5.5 |  |
| C6 | 649.2 | -73.7 |  | 74.3 | -2.8 |  | 8.1 | 4.0 |  |
| C7 | 327.2 | -28.0 |  | 99.3 | -23.1 |  | 18.0 | -2.7 |  |
| C8 | 402.3 | 42.2 |  | 74.3 | 2.8 |  | 18.0 | -3.9 |  |
| C9 | 121.0 | 11.4 |  | 131.4 | 23.1 |  | 28.6 | 2.0 |  |
| C10 | 562.0 | 14.2 |  | 47.9 | -17.5 |  | 12.8 | -6.6 |  |
| C6-1 | 649.2 | 31.5 |  | 42.4 | 0.0 |  | 8.1 | 4.1 |  |
| C6-2 | 768.3 | 12.3 |  | 15.8 | 1.6 |  | 8.1 | 3.2 |  |
